# Why Students Higher in Callous-Unemotional Traits Are More Resistant to Targeted Anti-Bullying Interventions by Teachers: The Role of Biased Perceptions

**DOI:** 10.1007/s10802-026-01450-1

**Published:** 2026-03-21

**Authors:** Claire F. Garandeau, Eerika Johander, Tiina Turunen, Christina Salmivalli

**Affiliations:** INVEST Flagship University of Turku, Assistentinkatu 7, Turku, 20500 Finland

**Keywords:** CU traits, Bullying, Anti-bullying intervention, Teacher intervention, Empathy, Blame

## Abstract

This study sought to understand why children high in callous-unemotional (CU) traits are less responsive to targeted anti-bullying interventions. We tested the effect of CU traits on intention to stop bullying after a teacher-led anti-bullying intervention and whether this effect was explained by participants’ perceptions of the teacher’s messages. A sample of 843 students in Grade 4 and 7 (49.8% boys, Mage = 11.56) was asked to imagine having bullied a peer and being invited to a meeting with a teacher. They were then shown a video depicting what the teacher would tell them in the meeting. They were randomly assigned to three conditions with different teacher messages (bullying-condemning, empathy-raising or a combination of both) and asked to rate the extent to which they perceived the teacher had condemned the bullying, tried to raise their empathy for the victim, and blamed them as a person. Analyses conducted separately for the whole sample and a subsample of bullying perpetrators revealed that higher levels of CU traits were associated with lower intention to stop bullying and with perceiving more blaming, less bullying-condemning and less empathy-raising from the teacher. These perceptions predicted lower intention to stop and partially mediated the effects of CU traits on intention to stop in the whole sample. In the subsample of perpetrators, only the indirect effect via perceived empathy-raising was statistically significant. One reason why youth high in CU traits are more resistant to anti-bullying intervention may be their biased perceptions of the content of anti-bullying messages.

When cases of bullying come to the attention of teachers, they have the moral – and often legal - responsibility to intervene and their interventions are generally associated with decreased victimization (e.g., Burger et al., 2022). Regarding the specific strategies that are the most likely to be effective, the current consensus is that both condemning the bullying behavior (a confronting approach) and attempting to raise the perpetrator’s empathy for the victim (a non-confronting approach) predict decreases in bullying behavior (Garandeau et al. 2014b) or a stronger intention to stop bullying (Garandeau et al., 2016; Johander et al., 2022), while blaming the perpetrator has no effect (Garandeau et al., 2016). However, teachers’ interventions do not always work (Johander et al., 2024). Some students are less responsive to anti-bullying interventions, whether it is to a whole-school program (Garandeau et al. 2014a; Nocentini et al. 2019) or to a targeted intervention (i.e., discussion with a teacher designed to put an end to the bullying; Johander et al., 2022). Unfortunately, personal characteristics that are relatively stable, such as temperament or personality traits, can predict such resistance (Nocentini et al., 2019). Callous-unemotional (CU) traits in particular have been shown to be associated with a lower intention to change behavior among students exposed to teachers’ anti-bullying message in a video-vignette experiment (Johander et al., 2022). High CU-trait children are also more resistant to teacher discipline strategies (Allen et al., 2018).

In order to make school anti-bullying efforts more effective, it is essential to understand why interventions fail (Salmivalli, 2023), which involves understanding why some children or adolescents are less responsive to anti-bullying interventions. Some studies have suggested that CU traits may be associated with biases in the processing of social information, more specifically with hostile attribution bias (Cima et al., 2014; Dapprich et al., 2024), less accuracy in the recognition of emotions (Ciucci et al., 2024) and impaired moral reasoning (e.g., Thornberg & Jungert, 2017). Therefore, it is possible that when children high in CU traits are exposed to the anti-bullying messages of teachers (e.g., empathy-raising, condemning of the behavior), their perception of these messages is distorted in such a way that they feel more blamed by the teacher and overlook their attempts to raise their empathy or their moral reprobation of the behavior. In turn, such perceptions could make them less likely to stop their bullying behavior.

We investigate these direct and indirect associations using data from an experimental study, in which participants watched a video of a teacher talking to them after they have supposedly bullied a peer. Participants were randomly assigned to three conditions; one in which the teacher focuses on condemning the bullying behavior, one in which the teacher attempts to raise the student’s empathy for the victim, and one which combines both strategies. Afterwards, they were asked to report the extent to which they felt that the teacher had condemned their bullying behavior, tried to arouse their empathy for the victim, blamed them personally, as well as their intention to stop their bullying. Analyses were conducted both in a general sample of children (Grade 4) and early adolescents (Grade 7) and in a subsample of participants who had been engaging in bullying behavior in real life.

## Effects of CU Traits on Intention to Stop Bullying

The term callous-unemotional (CU) refers to a combination of personality traits that include lack of empathy and remorse, disregard for others, and shallow or deficient affect (e.g., Viding et al., 2019). These traits tend to be relatively stable from early childhood to adulthood (Frick et al., 2003), probably because the genetic influence on CU traits is relatively strong (Moore et al., 2019; Viding & McCrory, 2012), although warm/rewarding parenting can moderate this genetic contribution (e.g., Henry et al., 2018). There is ample evidence of positive associations of CU traits with aggression in the classroom (Allen et al., 2016; Waschbusch et al., 2015) and bullying behaviors (Fanti et al., 2019; Thornberg et al., 2025; Van Geel et al., 2017; Zych et al., 2019).

Importantly, research indicates that children and youth with high levels of CU traits are less likely to be responsive to attempts at putting an end to their aggressive behavior. In early adolescence, scoring high on CU traits was found to be strongly correlated with punishment insensitivity, both self-reported (e.g., “You do not care when you are punished;” Allen et al., 2016; Allen et al., 2018; Hwang et al., 2021) and reported by teachers (Allen et al., 2016). The teachers participating in the study by Allen et al. (2018) were blind to the CU trait status of their students and – when interviewed - described students high in CU traits as resistant to teacher discipline strategies. Furthermore, among children and adolescents treated for conduct problems, high levels of CU traits have been linked to significant but limited treatment effectiveness (Hawes et al., 2014; Waschbusch et al., 2025).

In a study testing the effects of teacher interventions (targeted at bullying perpetrators) on students’ intention to stop bullying and investigating different messages delivered by the teacher (condemning the bullying behavior vs. raising empathy for the victimized peer), CU traits were negatively associated with the intention to stop (Johander et al., 2022). Consistent with the findings about youth with conduct problems, youth high in CU traits were less likely to respond to an intervention aimed at decreasing their bullying behavior, and CU traits did not moderate the relative effectiveness of different messages. Based on this literature, in the current study, we hypothesize that students with higher levels of CU traits will report a lower intention to change behavior compared to those with lower levels of CU traits.

### The Role of Cognitive Distortion in the Perception of Anti-Bullying Messages

One reason for the lower responsiveness of high CU-trait students to discipline attempts might be related to their processing of social information. It is well-documented that children high in CU traits tend to suffer from deficits in the recognition of several emotions, including fear and sadness (Ciucci et al., 2024; Dawel et al., 2012) and might have a tendency to mistake fear for anger (Ciucci et al., 2024). Among youth displaying antisocial behavior, CU traits are also associated with emotional attention deficits (Kimonis et al., 2018). When it comes to social situations specifically, there is evidence that CU traits are linked to hostile interpretation bias (i.e., interpreting ambiguous or neutral stimuli, such as tone or facial expressions, in a hostile or threatening way) among delinquent adolescents (Cima et al., 2014) and among adolescent inpatients suffering from various psychological disorders (Dapprich et al., 2024). In normative samples of children and adolescents, positive associations have also been found between having a hostile attribution bias (i.e., the tendency to attribute hostile intent to others’ neutral and ambiguous behaviors) and CU traits (Kokkinos & Voulgaridou, 2018) or only with the callousness dimension of CU traits (Voulgaridou et al., 2022). It is important to note that a positive link between CU traits and hostile attribution bias is not universally supported (e.g., Frick et al., 2003; Helseth et al., 2015) and that most studies examine this bias in response to peers rather than authority figures. Nonetheless, hostile attribution bias is generally considered as a relatively stable social–cognitive processing style and in that respect can be expected to influence individuals’ interpretation of social cues across various relational contexts. Therefore, these findings could suggest that when bullying perpetrators are exposed to an anti-bullying discussion by a teacher in their school, they might have a negatively distorted perception of the message if they are high in CU traits. Specifically, they might be more likely to attribute hostile intention to the teacher, and therefore more likely to feel that the teacher is blaming them personally.

CU traits might also affect children and youth’s perceptions of the extent to which the teacher tries to raise their empathy for the victim of bullying or condemns their bullying behavior. These two types of messages both implicitly refer to moral values, by emphasizing how wrong it is to bully others and how much suffering it causes to the victim. Both aim at arousing moral emotions, such as guilt (and there is evidence that they do; see Johander et al., 2025). Since CU traits – at least in combination with conduct problems - are often associated with deficiencies in moral reasoning (Northam et al., 2022), reduced physiological responses to emotional stimuli, including distress cues (e.g., Blair, 1999; de Wied et al., 2012), and deficiencies in attentional orienting to negative stimuli (e.g., Ciucci et al., 2018; Kimonis et al., 2018), it is possible that children high in CU traits perceive less strongly than others that the teacher expressed how wrong bullying is and tried to elicit empathy for the victim.

In turn, such distorted perceptions might make students more likely to report a desire to continue their behavior. Indeed, there is a robust positive link between hostile attribution bias and aggressive behavior (Orobio de Castro et al., 2002; Verhoef et al., 2019), suggesting that perceiving the teacher as more blaming might predict a lower intention to stop bullying. Furthermore, a recent study has shown that perceiving the teacher’s message in such discussions as less empathy-raising and as less condemning of the bullying was associated with a lower intention to stop bullying (Johander et al., 2025). Therefore, in the present study, we expect that higher levels of CU traits will be associated with a lower intention to stop bullying via negatively biased perceptions of the teacher’s message, namely higher perceived blaming, lower perceived empathy-raising and lower perceived bullying-condemning.

## Method

### Procedure and Participants

Between October 2024 and March 2025, data were collected from a convenience sample of 4th - and 7th -graders in 12 primary, 4 secondary, and 6 combined schools across Finland. In three of the combined schools, only students in the primary grades were included, and in one of the combined schools, only students in the secondary grades were included, in accordance with school decisions. The principals of these schools were informed about the study’s purpose and procedures and asked to invite all 4th- and 7th-grade students to participate. A few weeks prior to data collection, parents or guardians of the students received information about the study procedures and data protection and were asked to fill out an informed consent form for their child’s participation. All students who returned a signed parental consent form participated in a token lottery (two movie tickets per classroom) regardless of whether their parents consented to their participation. Only students who received parental consent and provided their own assent on the day of data collection participated in the study, which was approved by the Ethics Committee for Human Sciences of the University of Turku.

During data collection, participants first completed a survey assessing their age, gender, bullying behavior, and CU traits. They were then asked to imagine having bullied a peer and being invited to a discussion with a teacher. They were exposed to the hypothetical teacher intervention via a video vignette (half of the videos featured a male teacher; half a female teacher). Participants were instructed to listen carefully, as the video would be shown only once. There were three main versions of the video: In one, the teacher focuses on *condemning the bullying behavior*; in another, the teacher focuses on *raising empathy for the victim*; in the third one, both messages are *combined*. The length of the videos ranged from 36 to 63 s. In a previous study conducted with a different sample, validity analyses showed that each message was overall perceived as intended (Johander et al., 2022). In addition, each of these three versions was either framed with an empowering message or not^1^. This empowering condition was added to allow us to explore whether teachers expressing their belief in the student’s ability to change behavior would improve the effectiveness of the condemning and empathy-raising messages by promoting the student’s self-efficacy to change. This led to a total of 6 versions. Each classroom was randomly assigned to one of these six experimental conditions, with the exception of five classrooms in which students were divided between two different conditions. Additionally, due to scheduling constraints, a few students in one classroom participated in a different experimental condition than the rest of their class. To facilitate data collection for the schools, students from multiple classrooms assigned to the same condition (within the same school) were grouped together to form a single test group. After seeing the video, they completed a second survey measuring their perception of the extent to which the teacher had condemned their bullying behavior, attempted to arouse their empathy for the victimized student, or blamed them. They also indicated how likely they would be to stop bullying if they were to experience a similar discussion in real life.

Participants included 857 students (405 in Grade 4 and 452 in Grade 7) from 92 classrooms in 22 schools and were divided into 76 test groups. Thirteen students (from three classrooms belonging to two test groups) were excluded due to technical issues during the video presentation, and one student was excluded due to clearly patterned responses on the survey. All excluded students were 7th-graders. The final sample included 843 students (420 boys, 404 girls, 19 with missing information on gender; *M*age = 11.56, *SD* = 1.53) from 89 classrooms and 74 test groups.

Since each classroom was randomly assigned to one of the six conditions (condemning, empathy-raising, and combined message, each with either an empowerment frame or not), levels of CU traits were expected to be similar across conditions. However, ANOVAs and Tukey tests revealed one significant difference. In the behavior-condemning (with empowerment) condition, CU-trait levels were lower (*M* = 1.41) than in the combined (with empowerment) condition (*M* = 1.67; *p* = .012). Therefore, our analyses control for the type of message the participants were exposed to and for the presence of an empowerment frame.

### Measures

*Callous-unemotional traits.* Participants’ callous-unemotional traits were measured using The Inventory of Callous-Unemotional Traits (ICU; Frick, 2004). The ICU is a 24-item self-report scale used to assess three aspects of callous-unemotional traits in youth: callousness (e.g., *I do not feel remorseful when I do something wrong*), uncaring (e.g, *I apologize -“say I am sorry”- to persons I hurt*) and unemotional (e.g., *I do not show my emotions to others*). Answers were given on a 6-point scale ranging from 0 = strongly disagree to 5 = strongly agree. Mean scores were calculated for the total callous-unemotional traits scale (Ω = .86, excluding items 2 and 10 as recommended by Ray et al. 2016; also, in our data, these two items reduced the scale reliability).

*Intention to stop hypothetical bullying behavior.* Six items assessed participants’ intention to stop bullying behavior: *If I had been in this situation and the teacher would have talked to me like this*,* (a) I would stop bullying the classmate; (b) it would be unlikely that I would bully others in the future because of what the teacher said to me; (c) I would not bully others anymore after this discussion; (d) I would probably continue bullying after this (reverse coded); (e) what the teacher said would very likely influence how I treat others in the future; and (f) the teacher’s words would have a strong impact on my behavior.* Responses were given on a 6-point scale ranging from 0 = strongly disagree to 5 = strongly agree. The McDonald’s omega (see Hayes & Coutts, 2020) for these six questions was satisfactory (Ω = 0.81).

*Perceived condemning of the bullying behavior.* Three items assessed the extent to which participants felt that the teacher had condemned their bullying behavior: *(a) the teacher clearly mentioned that I have behaved wrongly; (b) the teacher told me that he/she knew that I had been bullying my classmate and demanded that I stop; and (c) the teacher held me responsible for the things that have happened.* Responses were given on a 6-point scale ranging from 0 = strongly disagree to 5 = strongly agree. The scores for the three items were averaged and the composite score had good reliability (Ω = 0.83).

*Perceived empathy-raising.* Four items assessed the extent to which participants felt that the teacher had tried to arouse their empathy towards the hypothetical victim: *(a) the teacher talked especially about how bad my classmate is feeling; (b) the teacher tried to make me understand how bad my classmate is feeling; (c) the teacher did not blame me*,* but wanted me to help the classmate who is having a difficult time; and (d) the teacher helped me to understand the difficult situation my classmate is in.* Responses were given on a 6-point scale ranging from 0 = strongly disagree to 5 = strongly agree. The scores for the four items were averaged and the composite score had good reliability (Ω = 0.76).

*Perceived person-blaming*. Two items assessed the extent to which the participants felt that the teacher had blamed them (as persons): *(a) the teacher said I was a mean person; (b) the teacher said that I don’t understand how others are feeling.* Responses were given on a 6-point scale ranging from 0 = strongly disagree to 5 = strongly agree. The scores for the two items were averaged. Their correlation was *r* = .50.

*Control variables.* The control variables used in the analyses were participants’ grade level (0 = grade 4, 1 = grade 7), gender (0 = girl, 1 = boy), the teacher speaking in the video (0 = female, 1 = male), the actual message in the video (condemning, empathy-raising, combined), the frame (empowerment message vs. not), as well as self-reported frequency of bullying in real life, which was assessed with four items. Students were asked to indicate whether they had bullied another student at school or online in any of the following ways in the last few months: *(a) called them bad names*,* made fun of them or treated badly; (b) purposely left them out or ignored them; (c) punched*,* kicked or pushed them; (c) told lies about them or tried to make others dislike them.* Responses were given on a 5-point scale (0 = not at all, 1 = only once or twice, 2 = two or three times a month, 3 = about once a week, and 4 = several times a week). The scores for the four items were averaged (Ω = 0.64). Although the measure did not explicitly assess repetition or power imbalance and may therefore have captured general aggressive behavior in addition to bullying, we hereafter refer to it as bullying for consistency with the intervention context. The relatively low reliability reflects the fact that engaging in one type of bullying (e.g., relational) is not necessarily associated with engaging in another type of bullying (e.g., physical). A subsample of bullying perpetrators was identified by selecting those who reported having bullied others at least once or twice (response 1). Although the cut-off that has been established for identifying bullies is two or three times a month (response 2), we chose a more lenient cut-off to ensure that this subsample (a) is large enough for our analyses and (b) includes those who under-report their level of bullying and anyone who is likely to be exposed to such discussions in real life (which includes infrequent bullies). In analyses conducted with this subsample, the frequency of bullying variable was mean-centered.

### Analysis Plan

The aim was to test whether perceptions of the anti-bullying messages (perceived condemning of the behavior, perceived empathy-raising and perceived blaming) mediated the association between CU traits and intention to stop bullying. Separate models were run for the full sample and for students who had engaged in bullying behavior in real life. All analyses controlled for the participants’ grade level, gender, self-reported frequency of bullying, the teacher speaking in the video (male or female), the video’s actual message (behavior-condemning, empathy-raising, or combined) and whether the video message was framed with an empowering message or not. First, the main effect of CU traits on intention to stop was tested (Model 1). Second, we examined the effects of perceived behavior-condemning, perceived empathy-raising, and perceived blaming on intention to stop (Model 2). Third, we examined the effect of CU traits on the perceptions of anti-bullying messages (Model 3a for perceived condemning, Model 3b for perceived empathy-raising, Model 3c for perceived blaming). Finally, Models 1, 2 and 3 were combined to examine the indirect effect of each perceived message in the association between CU traits and intention to stop bullying (Model 4). The hypothesized mediators were allowed to correlate. For all models, both unstandardized and standardized effects are provided.

Analyses were conducted using *M*plus 8.8 (Muthén & Muthén, 1998–2023) and maximum likelihood estimation (ML). The indirect effects were tested via bias-corrected 95% bootstrap confidence intervals based on 10,000 bootstrap draws. Direct and indirect effects were considered significant when the corresponding 95% confidence intervals did not include zero (Preacher & Hayes, 2004). Based on research assistants’ observations during data collection and a careful data inspection, scales in the second survey showed clearly patterned responding for three students, so their responses were coded as missing. Missing data was handled using full information maximum likelihood estimation (FIML). Clustering of participants within test groups was accounted for using the COMPLEX option, which adjusts standard error estimates to correct for non-independence of observations due to between-group variation. Indeed, situational factors during the testing sessions could lead to greater similarity in responses among students within the same test group. The intraclass correlation (ICC) of intention to stop bullying indicated that 9% of the total variance was attributable to differences between test groups.

## Results

### Effects of CU Traits and Perceived Messages on Intention to Stop Bullying

Correlations and descriptive statistics for the study variables are presented in Table 1. The main effect of CU traits on intention to stop bullying was tested in Model 1 (see Table 2). In the whole sample, higher levels of CU traits (*β* =-0.49), as well as higher frequency of bullying behavior (*β* =-0.16) were associated with a lower intention to stop bullying. In addition, Grade 4 students reported a higher intention to stop than Grade 7 students (*β* =-0.10). In the subsample of perpetrators, higher levels of CU traits were also associated with a lower intention to stop bullying (*β* =-0.55). In this subsample, receiving an empowering message (*β* = 0.13) and being in Grade 7 (vs. Grade 4; *β* = -0.11) were associated with a higher intention to stop bullying. No other predictor had a significant effect.

**Table 1:** Correlations and descriptive statistics of study variables

Variable	1	2	3	4	5	6	7	8	9	10	11	12	13	
1. Intention to stop bullying	-													
2. CU traits	− 0.55^***^	-												
3. Grade level	− 0.20^***^	0.21^***^	-											
4. Boy (student)	− 0.17^***^	0.26^***^	0.02	-										
5. Male (teacher)	0.01	0.01	− 0.03	0.06	-									
6. Frequency of bullying	− 0.28^***^	0.25^***^	− 0.00	0.16^***^	0.02	-								
7. Condemning	0.04	− 0.07	− 0.01	− 0.03	− 0.01	− 0.07^*^	-							
8. Empathy-raising	0.02	− 0.02	0.00	− 0.01	0.01	0.01	− 0.50^***^	-						
9. Combined	− 0.05	0.09^*^	0.01	0.04	− 0.00	0.06	− 0.49^***^	− 0.51^***^	-					
10. Empowerment	0.05	− 0.01	− 0.01	0.03	− 0.04	− 0.01	− 0.01	0.02	− 0.01	-				
11. Perceived condemning	0.09^**^	− 0.07^*^	− 0.04	0.05	0.03	0.01	0.30^***^	− 0.56^***^	0.27^***^	− 0.08^*^	-			
12. Perceived empathy-raising	0.31^***^	− 0.19^***^	− 0.10^**^	0.06	0.06	− 0.04	− 0.38^***^	0.42^***^	− 0.05	0.21^***^	− 0.15^***^	-		
13. Perceived blaming	− 0.19^***^	0.25^***^	− 0.11^**^	0.13^***^	0.05	0.10^**^	0.00	− 0.15^***^	0.15^***^	− 0.17^***^	0.31^***^	− 0.09^*^	-	
*M*	4.07	1.52	0.52	0.51	0.49	0.15	0.32	0.34	0.34	0.48	3.56	3.52	1.54	
*SD*	0.90	0.64				0.32					1.39	1.12	1.34	
Min	0.00	0.00				0.00					0.00	0.00	0.00	
Max	5.00	4.14				3.25					5.00	5.00	5.00	

*Note*. Correlation coefficients between binary variables are phi coefficients

***p <.001. **p <.01. *p <.05

**Table 2:** Effects of CU traits and perceptions on intention to stop bullying

	Whole sample (*N* = 843)								Subsample of bullying perpetrators (*N* = 247)					
	Model 1				Model 2				Model 1			Model 2		
Predictor		*b*	95% *CI*	*SE*		*b*	95% *CI*	*SE*	*b*	95% *CI*	*SE*	*b*	95% *CI*	*SE*
Grade level		**-0.17**	**[-0.28**,** -0.06]**	**0.06**		**-0.32**	**[-0.45**,** -0.18]**	**0.07**	**-0.21**	**[-0.41**,** -0.01]**	**0.10**	**-0.33**	**[-0.55**,** -0.12]**	**0.11**
Boy (student)		-0.02	[-0.14, 0.11]	0.06		**-0.22**	**[-0.34**,** -0.11]**	**0.06**	-0.12	[-0.35, 0.10]	0.12	**-0.32**	**[-0.54**,** -0.07]**	**0.12**
Male (teacher)		0.03	[-0.08, 0.15]	0.06		0.00	[-0.13, 0.14]	0.07	-0.02	[-0.21, 0.18]	0.10	-0.01	[-0.22, 0.21]	0.11
Frequency of bullying		**-0.45**	**[-0.65**,** -0.27]**	**0.10**		**-0.63**	**[-0.84**,** -0.43]**	**0.11**	-0.28	[-0.57, 0.01]	0.15	**-0.51**	**[-0.82**,** -0.20]**	**0.16**
Condemning		-0.01	[-0.16, 0.12]	**0.07**		**0.17**	**[0.02**,** 0.33]**	**0.08**	-0.16	[-0.40, 0.10]	0.13	0.04	[-0.24, 0.33]	0.15
Empathy-raising		-0.00	[-0.14, 0.13]	0.07		-0.05	[-0.25, 0.14]	0.11	-0.10	[-0.33, 0.11]	0.12	-0.15	[-0.46, 0.13]	0.15
Empowerment		0.09	[-0.02, 0.20]	0.06		-0.08	[-0.23, 0.06]	0.07	**0.26**	**[0.05**,** 0.46]**	**0.10**	0.10	[-0.13, 0.31]	0.11
CU traits		**-0.69**	**[-0.79**,** -0.60]**	**0.05**					**-0.82**	**[-1.01**,** -0.64]**	**0.10**			
Perceived condemning						**0.11**	**[0.05**,** 0.16]**	**0.03**				**0.13**	**[0.03**,** 0.24]**	**0.05**
Perceived empathy-raising						**0.28**	**[0.21**,** 0.35]**	**0.03**				**0.30**	**[0.17**,** 0.42]**	**0.06**
Perceived blaming						**-0.14**	**[-0.19**,** -0.09]**	**0.03**				**-0.16**	**[-0.26**,** -0.07]**	**0.05**
R^2^		**0.337**		**0.03**		**0.276**		**0.04**	**0.423**		**0.05**	**0.342**		**0.07**

*Note*. Significant effects are shown in bold. For the actual message variables (condemning and empathy-raising), the reference category is the combined category

The effects of perceived condemning, perceived empathy-raising and perceived blaming on intention to stop were tested in Model 2 (see Table 2). It included the same covariates as Model 1, except for CU traits. In both the whole sample and the subsample of perpetrators, perceiving the teacher’s message as more condemning of the bullying behavior (*β* = 0.17 and *β* = 0.18, respectively) and as more empathy-raising (*β* = 0.34 and *β* = 0.35) was associated with a higher intention to stop bullying, while perceiving more blaming was negatively associated with intention to stop (*β* =-0.20 and *β* = − 0.27). Moreover, older students (*β*s =-0.17), boys (*β* =-0.12 and *β* = − 0.17), and those reporting bullying others more often (*β*s =-0.22), reported lower intention to change. Being exposed to the bullying-condemning experimental condition (compared with the combined condition) predicted a higher intention to change behavior in the whole sample only (*β* = 0.09).

### Effects of CU Traits on the Perception of Messages

The effects of CU traits on each perceived message were tested in three models, one for each perceived message, and are presented in Table 3. In both the whole sample and the subsample of bullying perpetrators, CU traits were significantly associated with the perception of each message. Students higher in CU traits perceived less condemning of the behavior (*β* =-0.10 and *β* =-0.17, respectively), less empathy-raising (*β* =-0.20 and *β* =-0.23) and more blaming (*β* = 0.25 and *β* = 0.33).

**Table 3:** Effects of CU traits on perceived messages

	Model 3a			Model 3b			Model 3c		
	Perceived condemning			Perceived empathy-raising			Perceived blaming		
Predictor	*b*	95% *CI*	*SE*	*b*	95% *CI*	*SE*	*b*	95% *CI*	*SE*
	Whole sample (*N* = 843)								
Grade level	-0.04	[-0.24, 0.15]	0.10	-0.13	[-0.29, 0.03]	0.08	**-0.43**	**[-0.62**,** -0.25]**	**0.09**
Boy (student)	**0.21**	**[0.02**,** 0.39]**	**0.10**	**0.23**	**[0.09**,** 0.37]**	**0.07**	0.16	[-0.03, 0.36]	0.10
Male (teacher)	0.07	[-0.11, 0.24]	0.09	0.13	[-0.02, 0.29]	0.08	0.08	[-0.11, 0.25]	0.09
Frequency of bullying	0.09	[-0.17, 0.31]	0.12	-0.08	[-0.40, 0.19]	0.15	0.09	[-0.27, 0.55]	0.21
Condemning	0.05	[-0.16, 0.25]	0.10	**-0.60**	**[-0.81**,** -0.39]**	**0.11**	-0.20	[-0.43, 0.04]	0.12
Empathy-raising	**-1.62**	**[-1.83**,** -1.40]**	**0.11**	**0.69**	**[0.53**,** 0.85]**	**0.08**	**-0.49**	**[-0.70**,** -0.27]**	**0.11**
Empowerment	**-0.19**	**[-0.36**,** -0.02]**	**0.09**	**0.45**	**[0.30**,** 0.62]**	**0.08**	**-0.44**	**[-0.62**,** -0.26]**	**0.09**
CU traits	**-0.22**	**[-0.36**,** -0.08]**	**0.07**	**-0.36**	**[-0.47**,** -0.26]**	**0.05**	**0.52**	**[0.38**,** 0.66]**	**0.07**
R^2^	**0.33**		**0.03**	**0.32**		**0.04**	**0.14**		**0.03**
	Subsample of bullying perpetrators (*N* = 247)								
Grade level	-0.13	[-0.41, 0.16]	0.14	-0.03	[-0.34, 0.28]	0.16	**-0.35**	**[-0.71**,** -0.01]**	**0.18**
Boy (student)	**0.34**	**[0.03**,** 0.67]**	**0.16**	0.14	[-0.21, 0.42]	0.16	0.26	[-0.14, 0.65]	0.20
Male (teacher)	0.03	[-0.24, 0.30]	0.14	0.17	[-0.15, 0.49]	0.16	-0.08	[-0.44, 0.30]	0.19
Frequency of bullying	0.14	[-0.25, 0.50]	0.19	0.04	[-0.41, 0.41]	0.21	-0.03	[-0.60, 0.68]	0.32
Condemning	0.07	[-0.22, 0.35]	0.15	**-0.65**	**[-1.08**,** -0.18]**	**0.23**	-0.07	[-0.57, 0.39]	0.24
Empathy-raising	**-1.57**	**[-1.88**,** -1.23]**	**0.16**	**0.70**	**[0.39**,** 1.00]**	**0.16**	**-0.51**	**[-0.92**,** -0.05]**	**0.23**
Empowerment	-0.24	[-0.51, 0.02]	0.14	**0.45**	**[0.16**,** 0.77]**	**0.16**	-0.23	[-0.57, 0.14]	0.18
CU traits	**-0.34**	**[-0.58**,** -0.10]**	**0.12**	**-0.41**	**[-0.61**,** -0.18]**	**0.11**	**0.70**	**[0.42**,** 0.98]**	**0.14**
R^2^	**0.36**		**0.04**	**0.32**		**0.07**	**0.17**		**0.06**

*Note. *Significant effects are shown in bold. For the actual message variables (condemning and empathy-raising), the reference category is the combined category

With regard to how the experimental condition was associated with the perception of each message, results from both the whole sample and from the subsample of perpetrators show that being exposed to the empathy-raising condition predicted perceiving more empathy-raising (*β* = 0.29 and *β* = 0.30, respectively), less bullying-condemning (*β* =-0.55 and *β* =-0.57) and less blaming (*β*s =-0.17), than being exposed to the combined condition. Being exposed to the bullying-condemning condition predicted perceiving less empathy-raising (*β*s =-0.25) but not more blaming. In addition, 7th-graders perceived less blaming than 4th-graders *β* =-0.16 and *β* =-0.13). Boys perceived more bullying-condemning (*β* = 0.07 and *β* = 0.13) and, in the whole sample only, more empathy-raising than girls (*β* = 0.10).

### Indirect Effects of CU Traits on Intention to Stop Bullying Via Perceived Messages

**Fig. 1:**
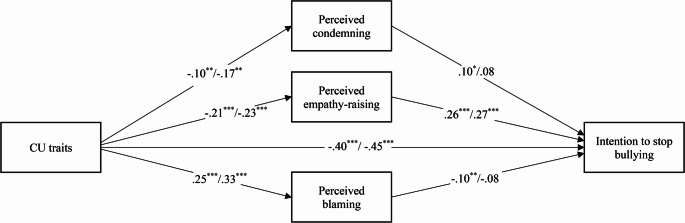
Standardized direct and indirect effects of CU traits on students’ intention to stop bullying. *Note.* Grade level, gender of the student, gender of the teacher, and self-reported frequency of bullying are controlled for, but not shown. Coefficients on the left of the dash are for the whole sample; coefficients on the right of the dash are for the subsample

Indirect effects of perceived messages on intention to stop bullying via emotions were examined in Model 4, which was built by combining Models 1, 2 and 3. The results of the full model with both direct and indirect effects are presented in Table 4 (unstandardized effects) and Fig. 1 (standardized effects). In the whole sample, there were significant indirect effects of CU traits on intention to stop bullying via less perceived condemning (*β*= − 0.01), less perceived empathy-raising (*β*= − 0.05), and more perceived blaming (*β*= − 0.03). The proportion of the association between CU traits and intention to stop explained by the mediator is 2.5% for condemning, 14% for empathy-raising and 7% for blaming. In the subsample of bullying perpetrators, the only indirect effect that reached significance was via perceived-empathy-raising (*β*= − 0.06), which explained 13.3% of that same association. The other indirect effects appeared only marginally significant, possibly due to a lack of power. In addition to these indirect effects, the direct effect of CU traits remained significant in the whole sample and among bullying perpetrators. The direct effects of each perceived message on intention to stop bullying also remained significant in the whole sample. In the subsample of perpetrators, only perceived empathy-raising positively predicted intention to stop. Moreover, intention to stop remained lower for participants in Grade 7 compared to Grade 4 and for participants reporting higher levels of bullying behavior.

**Table 4:** Direct effects of CU traits and message perceptions, and indirect effects of CU traits via perceptions on intention to stop bullying

	Whole sample (*N* = 843)			Subsample of bullying perpetrators (*N* = 247)		
	Model 4			Model 4		
Variable	*b*	95% *CI*	*SE*	*b*	95% *CI*	*SE*
Grade level	**-0.17**	**[-0.28**,** -0.06]**	**0.06**	**-0.21**	**[-0.41**,** -0.04]**	**0.10**
Boy (student)	-0.07	[-0.19, 0.05]	0.06	-0.15	[-0.37, 0.08]	0.12
Male (teacher)	0.00	[-0.11, 0.12]	0.06	-0.06	[-0.24, 0.11]	0.09
Frequency of bullying	**-0.44**	**[-0.61**,** -0.28]**	**0.08**	**-0.30**	**[-0.57**,** -0.03]**	**0.14**
Condemning	0.10	[-0.04, 0.23]	0.07	-0.01	[-0.26, 0.24]	0.13
Empathy-raising	-0.07	[-0.25, 0.09]	0.09	-0.20	[-0.47, 0.04]	0.13
Empowerment	-0.03	[-0.16, 0.09]	0.06	0.15	[-0.05, 0.34]	0.10
CU traits	**-0.57**	**[-0.67**,** -0.48]**	**0.05**	**-0.67**	**[-0.86**,** -0.49]**	**0.10**
Perceived condemning	**0.06**	**[0.02**,** 0.11]**	**0.02**	0.06	[-0.04, 0.15]	0.05
Perceived empathy-raising	**0.21**	**[0.15**,** 0.27]**	**0.03**	**0.23**	**[0.13**,** 0.33]**	**0.05**
Perceived blaming	**-0.07**	**[-0.12**,** -0.02]**	**0.02**	-0.06	[-0.15, 0.03]	0.05
Indirect effect via perceived condemning						
CU traits	**-0.01**	**[-0.04**,** -0.00]**	**0.01**	-0.02	[-0.07, 0.01]	0.02
Indirect effect via perceived empathy-raising						
CU traits	**-0.08**	**[-0.11**,** -0.05]**	**0.02**	**-0.09**	**[-0.18**,** -0.03]**	**0.04**
Indirect effect via perceived blaming						
CU traits	**-0.04**	**[-0.07**,** -0.01]**	**0.01**	-0.04	[-0.12, 0.01]	0.03
R^2^	**0.399**		**0.03**	**0.488**		**0.06**

*Note. *Significant effects are in bold. For the actual message variables (condemning and empathy-raising), the reference category is the combined category

### Additional Exploratory Analyses

Since exposure to the empowerment message had a significant positive effect on intention to change for bullying perpetrators, we decided to further explore whether this effect was moderated by CU-traits. However, the interaction was not significant either in the whole sample (*b* = − 0.12, *p* = .23) or among perpetrators (*b* = − 0.03, *p* = .87).

## Discussion

Although both whole-school anti-bullying programs and interventions targeted at bullies specifically have been shown to be effective in reducing bullying behavior and victimization, the effect sizes are usually modest (Gaffney et al., 2021) – especially in adolescence (see Salmivalli et al., 2021) - and these interventions do not work equally well with all students (Garandeau et al. 2014a; Johander et al. 2024; Nocentini et al. 2019). Personality characteristics, such as CU traits, have been shown in past research to be related, not only to higher levels of aggressive behavior, including bullying, but to a lower responsiveness to interventions designed to reduce problematic behaviors (e.g., Allen et al., 2018; Hawes et al., 2014; Hwang et al., 2021; Johander et al., 2022). The current study aimed to understand why, by testing whether students high in CU traits perceive the anti-bullying message of the teacher differently than other students and whether their perceptions, in turn, predict their intention to change. Our findings support this prediction.

### Do CU Traits Matter for Anti-Bullying Interventions’ Effectiveness and Why?

Consistent with our expectations and with prior literature, our findings indicate that CU traits matter for students’ responsiveness to anti-bullying interventions. Students scoring higher on CU traits reported lower intention to stop bullying after exposure to the teacher intervention, and this was the case both for the general sample of students and for those who reported that they had been engaging in bullying. This confirms that children and youth are not equally responsive to interventions designed to change their problematic behavior and that personal characteristics that are relatively stable, such as CU traits, can make them more resistant to adults’ anti-bullying efforts.

The main contribution of the present study is to demonstrate that one of the reasons why students high in CU traits are less likely to respond positively to anti-bullying interventions is that they do not perceive the teacher’s anti-bullying messages in the same way that students who are lower in CU traits do. Specifically, they perceived more blaming from the teacher, suggesting that they were more likely to interpret the teacher’s words as a personal attack or negative judgment on who they are. This is consistent with prior research showing a positive link between CU traits and hostile attribution bias (e.g., Kokkinos & Voulgaridou, 2018). Although several studies found that students high in CU traits displayed higher hostile attribution bias only when they exhibited conduct problems, such as delinquency (e.g., Cima et al., 2014), our study found evidence of a negatively biased interpretation of the message both among students who had engaged in bullying and in the whole sample. Perceiving more blaming might also be related to the fact that CU traits are positively correlated with narcissism (Jezior et al., 2016). Narcissistic individuals are known to have a need to be admired, a fragile self-esteem as well as tendencies towards paranoia, defined as the unfounded belief that another person intends to cause one harm (Freeman & Garety, 2014). These tendencies might lead them to interpret as personal attacks, or identity threat, what others simply see as behavioral critique. Thus, these adult interventions might activate in youth high in CU traits defensive responses to communication from authority figures, which likely undermines their internalization of the anti-bullying norm.

Not only were CU traits associated with perceiving more person-blaming, they were related to perceiving less behavior-condemning and less empathy-raising for the victim. In turn, these perceptions explained lower intention to change behavior. These findings are consistent with our predictions. We had reasoned that children and youth high in CU traits would be less receptive to these two messages, because they refer to moral values and appeal to moral emotions (e.g., guilt) and research suggests that youth high in CU traits, especially if they exhibit conduct problems, are deficient in moral reasoning (Northam et al., 2022), as well as less likely to respond to emotional stimuli at the physiological level (e.g., de Wied et al., 2012). These findings also advance theory on CU traits by showing that reduced responsiveness to anti-bullying interventions among students high in CU-traits may not only be due to deficits in empathy and moral motivation but to differences in how they perceive and interpret the anti-bullying messages. Rather than simply being morally disengaged, these students may systematically misinterpret moral cues, especially in adult communication. It is worth noting that the indirect effect via less perceived empathy-raising had the highest effect size compared to the other two perceptions and was the only significant indirect effect among perpetrators. It explained 13–14% of the association between CU-traits and intention to change, which corresponds in practice to a detectable, meaningful effect, especially given that the mediator is modifiable.

These findings have implications for teachers and other school personnel who address cases of bullying among their students. Since students high in CU traits may be more resistant to such interventions because of distorted – or at least biased relative to others - perceptions of the messages conveyed by teachers, the effectiveness of these interventions may be increased if teachers tailor their messages to these students. Their main goal should be to ensure that the student does not perceive excessive blaming by making the absence of personal blame more explicit. Instead, the teacher should emphasize that it is the behavior of the student that is problematic and that behaviors do not define individuals and can easily be changed. Adding guiding discussions designed to teach students to question their hostile attributions in ambiguous social situations (i.e., cognitive restructuring) might also be helpful (see Van Bockstaele et al., 2020).

### Limitations

The main limitation of the present study is that the targeted intervention was hypothetical. We used video vignettes depicting an adult talking to a student who had been bullying others, and participants were asked to imagine how they would feel and respond if they had been exposed to such a discussion after having bullied a peer at school. The use of these vignettes was to ensure that all students would receive the exact same message, regardless of their level of CU-traits, and our analyses were conducted both with the overall sample of students and with a subsample of students who had been engaging in bullying in real life. However, our findings may not generalize to actual interventions conducted after real-life bullying incidents. Moreover, the subsample of perpetrators may not have been large enough to detect some of the effects.

A second limitation concerns the fact that, despite the random assignment of classrooms to the various experimental conditions, average levels of CU traits were slightly higher in the combined condition (with empowerment) than in the bullying-condemning (with empowerment) condition. We took this limitation into account in our analyses by controlling for the actual condition that the participants were exposed to. Therefore, it is unlikely that our results would be biased by this unequal distribution. Finally, all measures were self-reported and therefore may be prone to social desirability bias. In particular, this might have led our group of “bullying perpetrators” to be smaller than it should have been, since those who engage in high levels of bullying tend to underreport their behavior (Garandeau et al., 2025). With regard to the bullying measure which captured four types of aggressive behavior, the consistency was quite low. However, since this variable was used mainly to identify a group of students who have engaged in bullying, treating each item separately would have been difficult (e.g., some engage in many forms) and was not highly relevant for the present study, since the anti-bullying discussion was designed to address any kind of bullying.

## Conclusion

Although research has been conducted to identify through which mechanisms and for whom whole-school anti-bullying programs are effective (Hensums et al., 2023), such knowledge is lacking for targeted anti-bullying discussions. The present study addressed this gap by showing that high levels of CU traits are associated with a lower willingness among children and youth to change one’s problematic behavior following a teacher intervention, and by offering an explanation why that might be the case: Students higher in CU traits appear to have a distorted perception of the messages issued by the teacher. Specifically, they perceive less empathy-raising and more person-blaming from the teacher. To a lesser extent, they perceived less condemning. In turn, these perceptions predict a lower intention to change. Therefore, these results suggest that addressing these biased perceptions, for example via cognitive restructuring exercises and by emphasizing the absence of personal blame, might improve the effectiveness of targeted anti-bullying interventions among these children.

## Data Availability

The data used in this study has not been deposited but is available upon request.

## Footnotes

1 This empowering message was: “I believe that you are someone who understands this situation and can be of help. Deep down you are a good person. I am sure you can make the school days nicer for him.”
